# Relative Selection Strength: Quantifying effect size in habitat‐ and step‐selection inference

**DOI:** 10.1002/ece3.3122

**Published:** 2017-06-14

**Authors:** Tal Avgar, Subhash R. Lele, Jonah L. Keim, Mark S. Boyce

**Affiliations:** ^1^ Department of Biological Sciences University of Alberta Edmonton AB Canada; ^2^ Department of Mathematical and Statistical Sciences University of Alberta Edmonton AB Canada; ^3^ Trove Predictive Data Science Edmonton AB Canada

**Keywords:** SSA, log odds, logistic regression, odds ratio, resource selection function, HSA, step selection function

## Abstract

Habitat‐selection analysis lacks an appropriate measure of the ecological significance of the statistical estimates—a practical interpretation of the magnitude of the selection coefficients. There is a need for a standard approach that allows relating the strength of selection to a change in habitat conditions across space, a quantification of the estimated effect size that can be compared both within and across studies. We offer a solution, based on the epidemiological risk ratio, which we term the relative selection strength (RSS). For a “used‐available” design with an exponential selection function, the RSS provides an appropriate interpretation of the magnitude of the estimated selection coefficients, conditional on all other covariates being fixed. This is similar to the interpretation of the regression coefficients in any multivariable regression analysis. Although technically correct, the conditional interpretation may be inappropriate when attempting to predict habitat use across a given landscape. Hence, we also provide a simple graphical tool that communicates both the conditional and average effect of the change in one covariate. The average‐effect plot answers the question: What is the average change in the space use probability as we change the covariate of interest, while averaging over possible values of other covariates? We illustrate an application of the average‐effect plot for the average effect of distance to road on space use for elk (*Cervus elaphus*) during the hunting season. We provide a list of potentially useful RSS expressions and discuss the utility of the RSS in the context of common ecological applications.

## 1 BACKGROUND

Habitat‐selection analysis (HSA) is central to many ecological studies and applications seeking to understand and/or predict the association between the probability of animal occurrence and local environmental conditions. Habitat‐selection studies often involve numerous habitat attributes and can be based on a variety of sampling and statistical‐modeling techniques. Consequently, appropriate interpretation and graphical presentation of the results and their ecological significance can be challenging, with consequences for effective communication of findings, as well as for the capacity to compare and synthesize across studies.

Broadly speaking, HSAs include two types of models that differ in their estimations procedure and hence in the type of predictions they generate. A “resource selection probability function” (RSPF) predicts the probability of selection of any given spatial unit (given its habitat attributes). In contrast, a “resource selection function” (RSF) yields prediction that are merely proportional to the probability of selection and hence cannot be directly used to project population abundance (see Boyce et al., 2016 for further discussion). Whereas the RSF is the most commonly used of the two models (for reasons explain below), for notational clarity we shall first present the RSPF. The typical functional form used in HSA is the exponential form:

(1) $$w\left(x\right)=c\cdot \exp\left[\sum_{i=1}^{p}\beta_{i}\cdot h_{i}\left(x\right)\right],$$

where *w*(*x*) is the value of the RSPF at position *x* in geographical space, β_*i*_ is the selection coefficient for the *i*’th habitat component, *h*_*i*_ (the *i*’th dimension in the *p*‐dimensional habitat space), and *c*[ = exp (β_0_), where β_0_ is the intercept] is a normalization factor ensuring that the function does not exceed 1. Note that, as *x* corresponds to a discrete unit in space (such as a map pixel or a habitat patch), it is appropriately termed a “resource unit” (Lele, Merrill, Keim, & Boyce, 2013). The theoretical justification for the use of this exponential form is that it is the discriminant function between two multivariate normal distributions (Manly, McDonald, Thomas, McDonald, & Erickson, 2002; Seber, 1984). Of course, habitat components can be a mixture of discrete and continuous variables, and hence, the joint normal distribution assumption for such a collection may be violated in many studies. Arguments have been made against the use of the exponential form for the RSPF due to the unreasonable parameter bounding it requires (Lele, 2009; Lele & Keim, 2006; McDonald, 2013).

The exponential form is nevertheless by far the most commonly used functional form in HSA. The majority of habitat‐selection studies are based on survey or telemetry approaches which inform us where animals are, but not necessarily where they are not, resulting in a “used‐available” (rather than a “used–unused”) design (Manly et al., 2002; McDonald, 2013). The prevalence of used‐available design is likely a key reason for the popularity of the exponential HSA, because under this design, the selection coefficients (i.e., the β_*i*_'s in Equation (1)) can be estimated using logistic regression, making it highly accessible (Johnson, Nielsen, Merrill, McDonald, & Boyce, 2006; McDonald, 2013). Under the used‐available design, however, the normalizing constant, *c*, in the exponential model, is nonidentifiable (Lele & Keim, 2006), and hence, inference can be drawn only about the relative probability of selection, resulting in an RSF rather than an RSPF. Note that considering the HSA results as yielding relative probability of selection, without mentioning the underlying exponential model, is misleading; if the underlying functional form is not of the type described in equation (1), such a blanket statement is incorrect.

One of the major, and as yet unresolved, problems in used‐available study design is the identification of available resource units, namely which resource units will be considered for use by the individual. A simplistic approach assumes that all resource units in the study area (often an arbitrary definition in itself) are equally considered for use if there is no selection. This has been modified to reflect the fact that not all resource units are equally encounterable. This leads to consideration of local availability that assumes all units in a small buffer around the previous location are available (e.g., Arthur, Manly, Mcdonald, & Garner, 1996; Baasch, Tyre, Millspaugh, Hygnstrom, & Vercauteren, 2010; Boyce et al., 2003; Compton, Rhymer, & McCollough, 2002; McCracken, Manly, & Heyden, 1998). This has been further modified to reflect the fact that limited availability arises due to movement limitations (Rhodes, McAlpine, Lunney, Possingham, & Centre, 2005), leading to the development of step selection analysis (SSA), where each “used step” (connecting two consecutive observed positions of the animal) is coupled with a set of “available steps,” randomly sampled from the empirical distribution of observed steps or their characteristics (Duchesne, Fortin, & Courbin, 2010; Forester, Im, & Rathouz, 2009; Fortin et al., 2005; Thurfjell, Ciuti, & Boyce, 2014). Lastly, a recent extension of SSA, termed integrated SSA (iSSA), allows explicit parameterization of a habitat‐independent movement kernel in conjunction with an HSA (Avgar, Potts, Lewis, & Boyce, 2016). SSAs allow incorporation of temporally dynamic covariates and can, thus, be used to test substantially more complex behavioral hypotheses than is possible using static‐availability HSAs (e.g., Fortin et al., 2005; Prokopenko, Boyce, & Avgar, 2017). Estimation of the parameters in the HSA under the local‐availability assumption (e.g., SSA/iSSA) is carried out using conditional logistic regression (case–control design), where each used location is coupled with, and contrasted against, a conditional availability set, sampled based on proximity in space and/or time. These models are, thus, computationally easy to fit. Whether one uses static availability (e.g., study area wide with no temporal dependencies) or dynamic availability (e.g., availability is defined by a movement kernel centered on the previously observed position), the basic HSA still relies on a used‐available design and an exponential selection function.

## 2 INTERPRETATION OF EXPONENTIAL HSA AND THE β Coefficients

Used‐available (whether static or dynamic) exponential HSAs allow the estimation of what is known in epidemiology as the “relative risk” or “risk ratio” (Miettinen, 1972). Relative risk is the ratio of the probability of an event occurring in a treatment group to the probability of the event occurring in a control group. Because we are working in the context of habitat selection, we shall refer to it as the relative selection strength (RSS).

### 2.1 Relative selection strength between two spatial locations

Let *x*_1_ and *x*_2_ denote the spatial coordinates of two locations. Then, *RSS* (*x*_2_, *x*_1_) = *w*(*x*_2_)/*w*(*x*_1_). Under the exponential model, this can be simplified as $RSS\left(x_{2},\;x_{1}\right)=\exp\left\{\Sigma_{i=1}^{p}\beta_{i}\left(h_{i}\left(x_{2}\right)-h_{i}\left(x_{1}\right)\right)\right\}$. Notice that this only depends on the difference in the habitat conditions between the two locations (or, in the case of an SSA/iSSA, the difference between two steps sharing the same starting point but ending in *x*_1_ and *x*_2_). Moreover, this does not depend on the normalizing parameter *c*[ = exp (β_0_)]. This ratio takes a value between 0 and ∞ and tells us which location, given that it is encountered, has a relatively higher probability of selection and by how much. There is a word of caution, however. Suppose there are four locations with *w*(*x*_1_) = 0.18, *w*(*x*_2_) = 0.90, *w*(*x*_3_) = 0.001, *w*(*x*_4_) = 0.005 as the selection probabilities. Then, RSS (*x*_2_, *x*_1_) = RSS (*x*_4_, *x*_3_) = 5. The RSS tells us that *x*_2_ is 5 times more probable than *x*_1_ but so is *x*_4_ five times more probable than *x*_3_. However, we would not treat those relationships to be equally important because the change in the first case seems ecologically far more important than in the second case.

### 2.2 Effect of a habitat covariate on selection

Aside from comparing two locations, in practice, we also want to know how the change in *one* of the habitat covariates will affect the probability of selection. This is a classic problem of interpretation of regression coefficients in multiple regression models. For example, we might want to interpret β_1_, the coefficient corresponding to the habitat covariate *h*_1_ in equation (1). Suppose we change the value of *h*_1_ by one unit and keep all other habitat covariates the same. Then, it is easy to see that $\frac{w(x;h_{1}+1,\;h_{2},\;h_{3},\ldots ,h_{p})}{w(x;h_{1},\;h_{2},\;h_{3},\ldots ,h_{p})}=\exp\left(\beta_{1}\right)$. Thus, exp(β_1_) is the RSS of habitat covariate *h*_1_, provided all other covariates in the model do not change. This is a conditional interpretation that is not the effect of the covariate *h*_1_without any reference to other covariates. Suppose we fit three different models; one with only *h*_1_, one with two covariates, *h*_1_ and *h*_2_, and one with three covariates *h*_1_, *h*_2_, and *h*_3_; as in any other multiple regression, the estimated coefficient corresponding to *h*_1_ in these three models, except in some rare situations, will be different (Seber, 1984). The inferred value of β_1_ and the interpretation of exp(β_1_)as the RSS is conditional on what other covariates are included in the model, what their values are, and whether these covariates are correlated or have correlated effects. Hence, this interpretation should not be thoughtlessly exported to other studies (but see below for a graphical methods for inference transferability).

## 3 COMMON RSS EXPRESSIONS

In our experience, certain statistical transformations and interactions are particularly common in HSA and SSA formulations. Here, we list the corresponding log‐RSS expressions in hope this will facilitate ease of use and interpretation. Note again that these are based on the assumption that all covariates not explicitly mentioned are kept constant.

**Figure 1:**
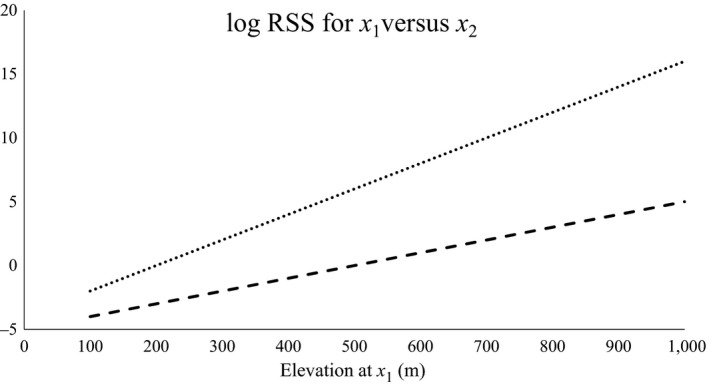
Log‐RSS for one spatial position (*x*_1_) over another (*x*_2_) as function of elevation and habitat type (“meadow” = dashed line; “forest” = dotted line) at *x*_1_. The RSF includes two main effects, one ctegorical (“forest”/“meadow”) and one continuous (elevation), as well as their interaction, and is given by exp(1·forest+0.01·elevation+0.01·elevation·forest). Elevation at *x*_2_ is 500 m, and habitat at *x*_2_ is “meadow” (the reference category for the RSF)
- If the HSA includes, in addition to *h*_i_, a squared term for $h_{i}\cdot \left(h_{i}^{2}\right)$, with a corresponding selection coefficient β_*i*2_, the conditional log‐RSS is given by $\Delta h_{i}\cdot \left(\beta_{i}+\beta_{i2}\cdot \left[2\cdot h_{i}\left(x_{1}\right)-\Delta h_{i}\right]\right)$. Hence, *in this case (and all subsequent cases), the log‐RSS is sensitive, in addition to the selection coefficients and the distance in habitat space,to the position in habitat space (i.e., the habitat value)*.
- In the combined case, where both a quadratic term and an interaction are included, the conditional log‐RSS is given by $\Delta h_{i}\cdot \left(\beta_{i}+\beta_{i2}\cdot \left[2\cdot h_{i}\left(x_{1}\right)-\Delta h_{i}\right]+\beta_{ij}\cdot h_{j}\left(x_{1}\right)\right)$.
- In the case where the habitat value is log‐transformed [ = ln (*h*_*i*_)], with a corresponding selection coefficient β_i_, the conditional log‐RSS is given by $\ln{\left[\frac{h_{i}\left(x_{1}\right)}{h_{i}\left(x_{1}\right)-\Delta h_{i}}\right]}^{\beta_{i}}$ (see Barrera‐Gómez & Basagaña 2015 for further discussion of log‐transformed variables). Hence, log‐transformed variables mean that the relative selection strength is a function of the ratio, rather than the difference, between available habitat values.
- In the case where the habitat value is log‐transformed, and there is an interaction with a second habitat component, *h*_j_, with a corresponding selection coefficient β_*ij*_, and given that $h_{j}\left(x_{1}\right)=h_{j}\left(x_{2}\right)$, the conditional log‐RSS is given by $\ln{\left[\frac{h_{i}\left(x_{1}\right)}{h_{i}\left(x_{1}\right)-\Delta h_{i}}\right]}^{\left[\beta_{i}+\beta_{ij}h_{j}\left(x_{1}\right)\right]}$.
- Lastly, in the case where two covariates, *h*_*i*_ and *h*_*j*_, are log‐transformed, the conditional log‐RSS for *x*_1_ in relation to *x*_2_ is given by $\ln{\left[\frac{h_{i}\left(x_{1}\right)}{h_{i}\left(x_{1}\right)-\Delta h_{i}}\right]}^{\beta_{i}}+\ln{\left[\frac{h_{j}\left(x_{1}\right)}{h_{j}\left(x_{1}\right)-\Delta h_{j}}\right]}^{\beta_{j}}$.

- The log‐RSS for location *x*_1_ in relation to location *x*_2_, given that these two locations share the same values for all habitat covariates but one, *h*_*i*_, is β_*i*_∙Δ*h*_*i*_, where Δ*h*_*i*_ = *h*_*i*_(*x*_1_) − *h*_*i*_(*x*_2_). For (i)SSA, *x*_1_ and *x*_2_ are further assumed to mark the end points of two steps starting from the same point in space and time (and hence sharing the same availability domain) and equal in their length (and any other attribute of the underlying movement kernel). In other words, β_*i*_ is the conditional log‐RSS over a unit distance in habitat space. If the two locations differ by two habitat components, *h*_i_ and *h*_j_, the conditional log‐RSS is β_*i*_∙Δ*h*_*i*_ **+ β_*j*_∙Δ*h*_*j*_, etc. Hence, *in these simple cases, the conditional RSS is sensitive only to the selection coefficients and the difference in habitat values (distance in habitat space), but not to the absolute value of the habitat*.
- If the HSA includes an interaction between *h*_*i*_ and *h*_*j*_ (*h*_*i*_·*h*_*j*_), with a corresponding selection coefficient β_*ij*_, and given that *h*_*j*_(*x*_1_) = *h*_*j*_(*x*_2_), the conditional log‐RSS is given by $\Delta h_{i}\cdot \left(\beta_{i}+\beta_{ij}\cdot h_{j}\left(x_{1}\right)\right]$ (see Figure 1).

## 4 AVERAGE EFFECT OF A HABITAT COVARIATE

The conditional interpretation of exp(β_1_) (i.e., the RSS) may be difficult to use when attempting to predict the intensity of space use as function of a focal covariate across a particular study area or management unit, where other covariates may, or may not, change in conjunction with the focal covariate. What one can, however, study is the *average change in the response as we change h*_1_ *, averaged over all possible values of other covariates in the model*. This interpretation still depends on what other covariates are present in the model, but it removes the problem of interpretation in the presence of correlations and conditioning on all other covariates not changing. This idea can be applied even in the case of an RSPF model, where the absolute probability of selection is computed. The mathematical details underlying this idea are presented in the Appendix.

Before proceeding further, we discuss the relationship between the RSS and the average effect depicted in the graphical tool. The probability of use is equivalent to the average probability of selection, averaged over all available units (Lele et al., 2013). Such an averaging weighs the probability of selection of a habitat type with the probability of encountering that habitat type. For a given probability of selection, higher encounter rate leads to higher probability of use, and inversely, lower encounter rate leads to lower probability of use (see Keim, DeWitt, & Lele, 2011). The graphical tool we describe here depicts the change in the *average* probability of selection as we change one of the habitat covariates while averaging over other habitat covariates according to their availability. Because we have averaged the selection probability over available resource units, this depicts the *change in the probability of use*, and not the change in the probability of selection. As will be illustrated below, if the availability of other resources changes, the graph depicting the probability of use also changes.

## 5 VISUALIZING THE AVERAGE EFFECT OF DISTANCE TO ROAD ON ELK SPACE USE

We offer an example illustrating the graphical method to help visualize and interpret the resource selection models, intended to demonstrate how to interpret graphical effect plots from resource selection studies. A detailed ecological analysis of the dataset is provided elsewhere (D. S. Ouren and J. L. Keim, unpublished manuscript).

Resource managers have identified hunting and off‐highway road management as necessary tools for elk management. Telemetry data on female elk were collected in western Colorado, USA, to document the influence of off‐highway roads on elk resource selection. Elk habitat‐use locations were collected using Global Positioning System (GPS) radiocollars deployed on 31 female elk between January 2006 and April 2009. In this analysis, we use a subset of the elk telemetry data to illustrate the graphical tools. The data include 5,686 elk habitat‐use relocations captured during the fall hunting season and 13,652 randomly distributed points (available locations) situated within 3 km of off‐highway roads. The analysis considers two covariates: habitat suitability index and distance to road (km). The habitat suitability index for any location was calculated based on a *separate* RSPF model fitted to an independent dataset on elk habitat‐use collected in the surrounding area. This RSPF model did not include distance to road as a covariate. The habitat suitability covariate, thus, stands as a proxy for including several habitat covariates such as terrain measures (e.g., slope, elevation, and aspect) and vegetation indices (e.g., normalized difference vegetation index).

We used the *ResourceSelection* package in R to estimate both the exponential RSF and the logistic RSPF models (Lele & Keim, 2006; Sólymos & Lele, 2016) from the data (Table 1 and 2). This package is readily available from CRAN (https://cran.r-project.org/web/packages/ResourceSelection/index.html) and can be applied using standard framework in R, similar to fitting a generalized linear model. Both the exponential and logistic models included an interaction effect between habitat suitability and road distance. We do not intend to discuss the model selection and appropriateness of different models, so only in passing we note that, based on the AIC, the logistic RSPF model had a better fit to the data than the exponential RSF model (AIC difference −415.826).

**Table 1:** Exponential resource selection function model

Parameter	Parameter estimate	*SE*	*Z*‐Value	Pr(>|*Z*|)
Habitat suitability	3.135	0.092	34.129	<2e‐16
Road distance	0.428	0.034	12.693	<2e‐16
Habitat suitability: Road distance	−0.406	0.053	−7.594	3.1e‐14

**Table 2:** Logistic resource selection probability function model

Parameter	Parameter estimate	*SE*	*Z*‐Value	Pr(>|*Z*|)
Intercept	−3.016	0.105	−28.697	<2e‐16
Habitat suitability	4.591	0.368	12.472	<2e‐16
Road distance	0.039	0.082	0.483	0.629
Habitat suitability: Road distance	3.030	0.452	6.708	1.98e‐11

### 5.1 Average effect of distance to road, averaged over all habitat conditions other than the distance to the road

To visualize the average effect of distance to road on the probability of space use by elk, we conducted the following analysis.

1. Fit the exponential RSF (or, logistic RSPF) model using two covariates; habitat suitability index and distance to road.
2. Compute the fitted exponential RSF (or, logistic RSPF) values at the available locations, namely {*w*(*x*_1_), *w*(*x*_2_), …, *w*(*x*_*N*_)}.
3. Plot the points {*h*_1_(*x*_*i*_), *w*(*x*_*i*_); *i* = 1, 2, …, *N*} where *h*_1_(*x*) is the distance to road for location *x*.
4. Use the function *ksmooth* in R to fit a smooth nonparametric regression function through these points.

**Figure 2:**
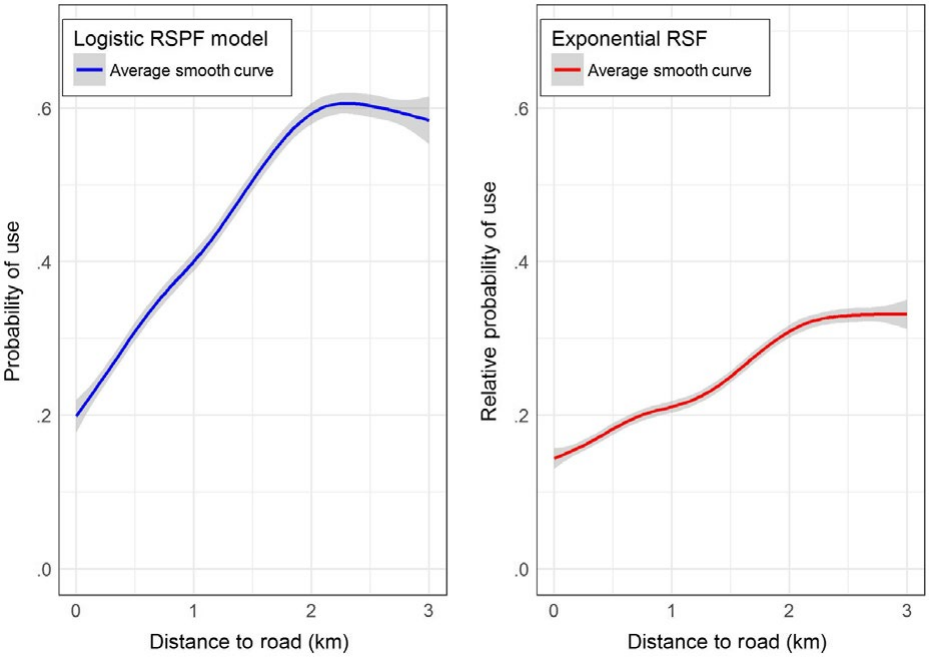
Average effect of distance to road on elk space use estimated from a logistic RSPF model and an exponential RSF model in the available distribution. The solid lines depict the smoothed nonparametric regression function between distance to road and the estimated probability of use or relative probability of use; 95% confidence intervals are depicted in gray shading

Each point on this smooth curve depicts the average RSF (or, average RSPF) for a given distance to road (*x*‐axis), where average is taken over habitat suitability index of all the available locations. The distribution of habitat suitability index over the available locations is the “available distribution for habitat suitability” in our study area. Hence, the function represents how space use by elk (rather than selection per se) changes as distance to road changes. Figure 2b depicts the change in the (relative) probability of use (as estimated by the exponential RSF), and Figure 2a depicts the probability of use (as estimated by the logistic RSPF) across the study area that lies within 3 km of off‐highway roads. The logistic RSPF model shows a much steeper relationship between the probability of space use by elk and distance to road as compared to the exponential RSF model.

**Figure 3:**
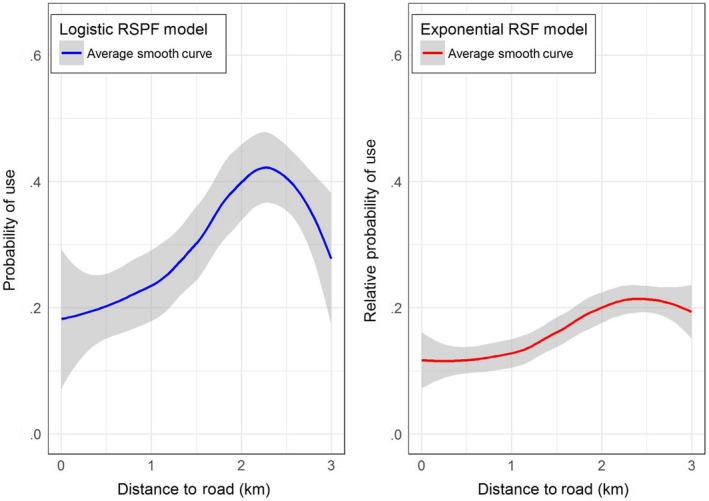
Average effect of distance to road on elk space use estimated from a logistic RSPF model and an exponential RSF model in a hypothetical study area. The solid lines depict the smoothed nonparametric regression function between distance to road and the estimated probability of use or relative probability of use; 95% confidence intervals are depicted in gray shading

Suppose now we want to use the fitted model to predict the probability of space use by elk in a different study area. This is useful, for example, to evaluate potential effects of habitat management strategy when adequate habitat‐use data are not available in the new area. To show how one can visualize the probability of space use by elk in a different study area, we generated a hypothetical study area that has a different available distribution of habitat suitability index and road distance conditions. The covariate composition in the hypothetical study area was generated using the following procedure. For any pixel, we randomly generated habitat suitability index values between 0 and 1 using a uniform distribution on (0,1), and distance to the road values was generated using an exponential distribution with mean 1, truncated at 3. We then predicted the estimated RSF and RSPF models across this hypothetical study area and plotted the average effect of road distance following the steps outlined above. In the case of the hypothetical study area (Figure 3), the probability of space use by elk is not as strongly influenced by road distance as it was in the original study area (Figure 2). Even though the resource selection model was unchanged, the result is different because of the effect of the specific spatial configuration of the new study area. Such a result should be expected when extrapolating any resource selection model across different management areas.

### 5.2 Preference curves for distance to road

**Figure 4:**
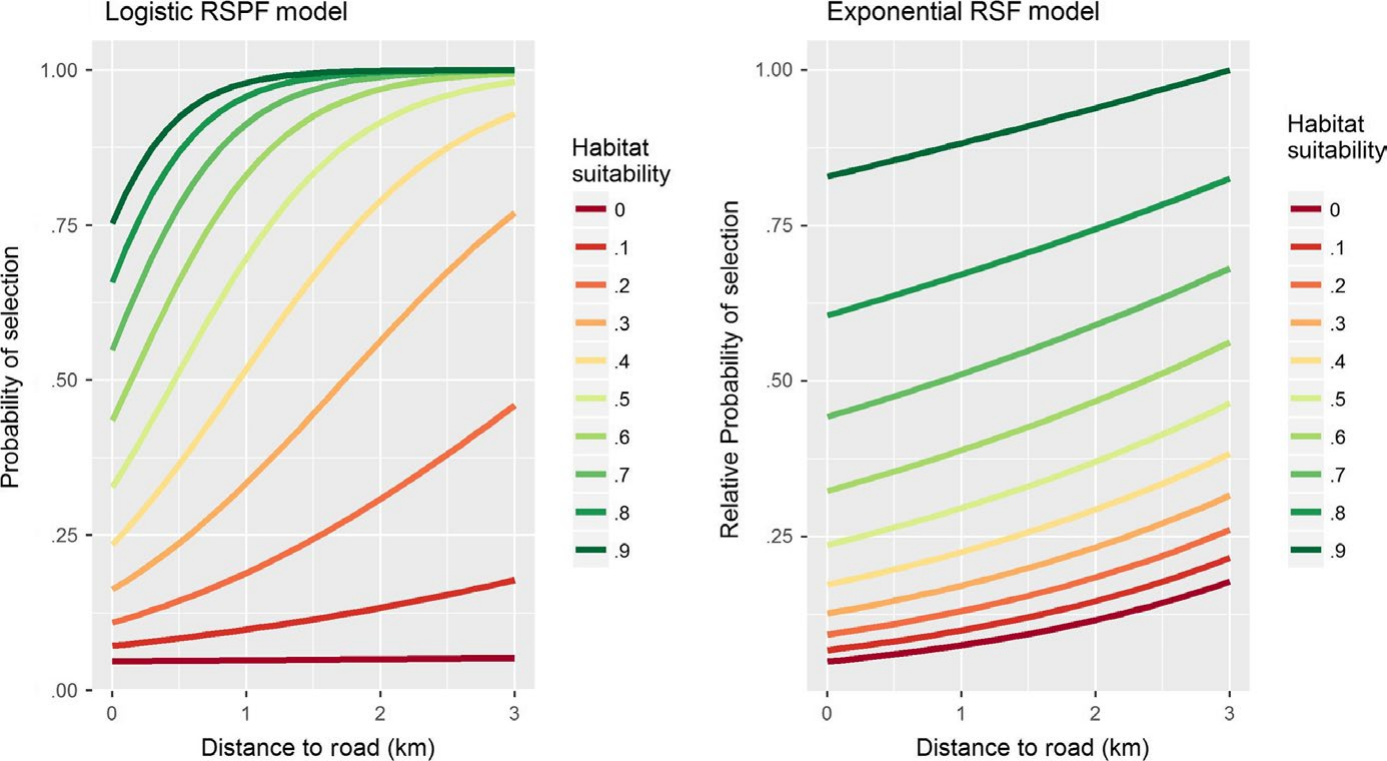
Resource selection preference curves for elk estimated from a logistic RSPF model and an exponential RSF model. The preference curves depict the estimated probability of selection (or relative probability of selection) assuming the distribution of road distance and habitat suitability resources are uniformly distributed and equally available to elk

Johnson (1980) suggests the term “preference” for “use when all resource types (not resource units), are encountered with equal probability.” In this specific case, “use” and “selection” functions turn out to be identical to each other. Borrowing from this concept, we can visualize the effect of a single covariate on the selection mechanism by considering a uniform distribution on the resource types (not resource units) as the available distribution and plot the average‐effect plot under this available distribution. Any specific study area will necessarily have different proportions in which different resource types are available. However, one can artificially impose a uniform distribution on different resource types and plot the average (or percentile) effect curves described earlier. We call the resultant plot “preference curves.” These plots enable one to see if there is any behavioral difference between elk from different geographic regions. Figure 4a,b depicts the preference curves for the exponential RSF and logistic RSPF models.

## 6 DISCUSSION

In this article, we have described the correct interpretation of the regression coefficients in the exponential RS(P)F model that is commonly used in HSAs. Binomial regression with logistic link is used to fit the exponential RSF to used‐available data. This has led some researchers to interpret the regression coefficients (β's) as log‐odds ratio as is used for logistic regression. We emphasize here again (see also, Lele et al., 2013) that this interpretation is incorrect. Although a binomial GLM is used to estimate the parameters, it is used only for computational purpose. The model being fit is still the exponential RSF (Equation (1) but with a nonidentifiable normalizing constant), and hence, the regression coefficients are correctly interpreted as “relative risk” or “relative selection strength” as described in this article. This is also true for local‐availability formulations (such as the iSSA) where conditional logistic regression is used only for computational purpose and the model being fit is still the exponential RSF.

All statistical analyses are based on assumptions (Sólymos & Lele, 2016). There are some strong assumptions that underlie the exponential HSA. For example, in SSA or iSSA, it is assumed that the set of covariates that affect movement are separate from the covariates that affect selection (Avgar et al., 2016; Forester et al., 2009). This assumption is unlikely to be true in practice. For example, movement and selection both are affected by vegetation type. Covariates that affect selection likely interact with covariates that affect movement. This assumption can be relaxed using the RSPF condition in Lele and Keim (2006) and Sólymos and Lele (2016). The main point, however, is that there are fairly strong assumptions underlying the HSA, assumptions that should be clearly stated when presenting the results. It is important to note moreover that there are no model diagnostic tools that we are aware of that can assure the researcher whether the assumptions about the available distribution, the selection‐free movement kernel or the exponential form of the RS(P)F are satisfied or not, but the results are strongly dependent on these assumptions.

Another type of study design, the used–unused study design, is sometimes used in HSA. Under this study design, we know the status, used or unused, of each resource unit in the study area. This type of study design can answer the question: What is the probability that a resource unit is used and how it depends on the habitat covariates? However, whether a resource unit is used or unused does not depend solely on its habitat characteristics and the selection strength but it also depends on other factors, mainly how many individuals are present in the study area. If the population size is large, available habitats are sampled (by the population) more intensely, and it is thus quite likely that even undesirable resource units are used and vice versa, if the population size is small, even highly desirable resource units may remain unused (Lele, Moreno, & Bayne, 2012). Note that, unlike the biological/behavioral effect of population density on selection strength (density‐dependent selection, for example, McLoughlin, Morris, Fortin, Vander Wal, & Contasti, 2010), the effect of population density on the probability of use is a statistical (sampling) effect, arising without any change in the underling selection function. The data from used–unused, or equivalently occupied–unoccupied, study design are useful to study the probability of occupancy but is not informative about the probability of selection. In practice, too many researchers equate probability of occupancy with probability of selection. This is incorrect. As was argued in Lele et al. (2013), probability of selection is different than probability of use. Whether a resource unit will be used or not depends on two factors: Would it be encountered? And, if encountered, would it be selected? This is why in SSA or iSSA, the encounter probability is modeled by the selection‐free movement kernel and probability of selection is modeled separately.

The fundamental difference between HSA and SSA is their respective definitions of the availability domain—the geographical space that is deemed accessible to the animal (and hence also the habitat space deemed available) at any point in space and time. In fact, different definitions of availability are common within “global” (unconditional) HSAs, ranging from a minimum convex polygon encompassing all observed occurrences (with or without buffers), through various types of kernel estimators (with various cutoff values), and on to the “population range” or simply the “study area” (Beyer et al., 2010; Prokopenko, Boyce, & Avgar, 2017 and refs therein). Not only that the resulting inference is sensitive to the definition of the availability domain (Beyer et al., 2010; Prokopenko, Boyce, & Avgar, 2017), it is often sensitive to the habitat availability and configuration within this domain (a so called “functional response”; Matthiopoulos, Hebblewhite, Aarts, & Fieberg, 2011; Mysterud & Ims, 1998; Paton & Matthiopoulos, 2016). We believe this point is also well reflected in our Figures 2, 3, 4. Consequently, care must be taken when comparing HSA inference across individuals or populations differing in their defined availability domains, and/or in the landscape composition within these domains.

A common practice in many HSA studies is to communicate the results via habitat‐selection maps, where the “selection” value in each map pixel, *x*, is calculated as: $\exp[\Sigma_{i=1}^{n}\beta_{i}\cdot h_{i}\left(x\right)]$. As can be seen based on our above definition of the RSS, these “selection” values are in fact the RSS in relation to a *reference pixel where all habitat values are zero*. A more useful map, perhaps, could be based on using a more typical pixel as a reference pixel. Then, the map can be interpreted in terms of relative selection strength, relative to the typical environmental conditions. Habitat‐selection maps, when based on a correctly formulated static‐availability HSA (where all relevant covariates are included and the availability domain is appropriately defined), are proportional to the expected probability density of use across the landscape, AKA, the utilization distribution (Avgar et al., 2016). Note, however, that this is not the case for a similarly derived SSA‐based map. In fact, the RSS aids in the correct interpretation of such SSA‐based maps; the pixel value is *the RSS of a step ending at that pixel in relation to an identical step (in terms of preceding steps, displacement and orientation) ending in a pixel where all habitat values are zero*. Such maps are thus less intuitive and useful than their “global” HSA‐based parallels. A utilization‐distribution map only can be derived based on (i)SSA by obtaining the steady‐state solution of the resulting stochastic matrix (the matrix of transition probabilities between each pair of pixels in the landscape), a task that might be computationally infeasible if the movement process includes velocity autocorrelation (and is hence a Markov process of order > 1). A Monte Carlo approximation of the steady state can be obtained by repeatedly simulating movement trajectories across the landscape raster based on the parametrized step‐selection function (Avgar et al., 2016; Signer, Fieberg, & Avgar, 2017).

The RSS offers a straightforward and easily interpretable measure of the conditional magnitude of the effect of any given habitat component, and we recommend its use in communicating and interpreting HSA and SSA results. From a management perspective, average‐effect plots and RSS maps are important tools in understanding and planning landscape changes and their potential effects on animal distribution and viability. Habitat‐selection studies often include numerous covariates relating to a variety of ecological effects potentially operating at a variety of scales. This complexity begets difficulties in interpreting and communicating findings, particularly the ecological significance of the effects. The RSS quantifies the relative strength of selection as function of the difference in habitat values, making it ideal as a measure of effect size. Specifically, for effective communication of HSA/SSA findings, we recommend plotting the log‐transformed RSS (so that negative values represent avoidance, whereas positive values represent selection) as a function of the difference in the value of one habitat component while keeping all other components constant (e.g., Figure 1 and Prokopenko, Boyce, & Avgar, 2017). For more complicated scenarios (e.g., transformations or interactions), the RSS may also be a function of the absolute habitat value, leading to 3D plots or multiple curves within the same plot. We believe such RSS plots should facilitate better understanding of the relative importance of various effects as well as comparisons across different studies.

## CONFLICT OF INTEREST

None declared.

## APPENDIX 1 MAPPING THE AVERAGE USE DISTRIBUTION

### 1.1

Average effect of a covariate

For the sake of simplicity, let us assume there are two covariates in the model, *h*_1_ and *h*_2_, along with interaction between them. Then, the exponential RSF model can be written as: *w*(*x*) = exp (β_1_ *h*_1_(*x*) + β_2_ *h*_2_(*x*) + β_12_ *h*_1_(*x*)*h*_2_(*x*)). We have provided the interpretation of the coefficients as the (conditional) relative selection strength. This interpretation is conditional, conditioned on all other covariates remaining the same. For most quantitative ecologists (and, indeed even for statisticians), this is difficult to visualize and interpret. Suppose we compute the RSF values at each of the *N* resource units (e.g., patches or map pixels) in our study area. That is, we have a set of values $\left\{w\left(x_{1}\right),w\left(x_{2}\right),\ldots ,w\left(x_{N}\right)\right\}$. Without loss of generality, we can standardize these values by dividing by their maximum. Thus, all values will be between 0 and 1. These values, however, do *not* correspond to probability of selection. These are still relative probabilities of selection, relative to the ‘most suitable resource unit’. We are interested in understanding the effect of habitat covariate *h*_1_ on the RSF. Toward this goal, we plot the points $\left\{h_{1}\left(x_{i}\right),w\left(x_{i}\right);i=1,2,\ldots ,N\right\}$. A nonparametric smooth of this plot mathematically corresponds to $\int \exp(\beta_{1}h_{1}\left(x\right)+\beta_{2}h_{2}\left(x\right)+\beta_{12}h_{1}\left(x\right)h_{2}\left(x\right))g\left(h_{2}|h_{1}\right)dh_{2}$ where $g(h_{2}|h_{1})$ is the distribution of habitat characteristics $h_{2}$ conditional on $h_{1}$ . This is the average effect of *h*_1_ on the RSS, averaged over the distribution of all values of the other covariates in the study area. Because we have averaged the probability of selection over the available distribution of the rest of the covariates, in the RSF context, this corresponds to the average relative use (relative to the most suitable resource unit) of any resource unit having first habitat covariate value $H_{1}=h_{1}$ . This, thus, depends on the specific configuration of the covariates in the study area. Note that a different spatial configuration of the covariates (with the same RSF model but a different $g(h_{2}|h_{1})$ or even a different *h*_2_range) will yield a different plot. It will tell us how the space use is affected by the habitat covariate $h_{1}$ in that specific spatial configuration of the resources. Thus, this plot answers the question: What is the predicted relative use distribution in a new study area if we control or change habitat covariate $h_{1}$?

If we use the weighted distribution approach described in Lele (2009) and Lele and Keim (2006), one can estimate the absolute probability of selection, assuming the RSPF condition (Lele & Keim, 2006; Sólymos & Lele, 2016) is reasonable. The interpretation of the average effect is significantly easier in this situation. Let us assume we are fitting a logistic RSPF, that is, $w\left(x\right)=\frac{\exp(\beta_{0}+\beta_{1}h_{1}\left(x\right)+\beta_{2}h_{2}\left(x\right)+\beta_{12}h_{1}\left(x\right)h_{2}\left(x\right))}{1+\exp(\beta_{0}+\beta_{1}h_{1}\left(x\right)+\beta_{2}h_{2}\left(x\right)+\beta_{12}h_{1}\left(x\right)h_{2}\left(x\right))}$. The interpretation of $\beta_{1}$ for this model is the same as in any logistic regression analysis. It is the change in the log‐odds of selection as we change the covariate by one unit, conditional on all other covariates remaining the same. Interpreting log‐odds is extremely difficult (see, e.g., Ramsey and Schafer, 2002, page 538–539). However, we can interpret the average effect on probability of use quite easily. As before, given the estimated model, we can compute $\left\{w\left(x_{1}\right),w\left(x_{2}\right),\ldots ,w\left(x_{N}\right)\right\}$ . These values do correspond to the probability of selection and lie between 0 and 1. We can, then, plot $\left\{h_{1}\left(x_{i}\right),w\left(x_{i}\right);i=1,2,\ldots ,N\right\}$. A nonparametric smoother through this plot corresponds to the average effect of *h*_1_ on the probability of selection, averaged over the distribution of all values of the other covariates in the study area. Because probability of selection is averaged over the available distribution, this is no more probability of selection (Lele et al., 2013), it is the probability of use corresponding to the particular covariate configuration in the study area. As in the RSF case above, a different spatial configuration corresponding to a new study area, but with the same RSPF model, will yield a different plot showing how probability of use will be affected by the habitat covariate $h_{1}$ in that specific study area. Instead of plotting the mean curve, one can also plot curves corresponding to different percentiles using the quantile regression methodology (Cade & Barry, 2003). Such curves, for example, will show the effect on space use on at least 75% of the resource units, etc.
